# The SLC9C2 Gene Product (Na^+^/H^+^ Exchanger Isoform 11; NHE11) Is a Testis-Specific Protein Localized to the Head of Mature Mammalian Sperm

**DOI:** 10.3390/ijms24065329

**Published:** 2023-03-10

**Authors:** Cameron C. Gardner, Paul F. James

**Affiliations:** Department of Biology, Miami University, Oxford, OH 45056, USA

**Keywords:** Na^+^/H^+^ exchangers (NHEs), SLC9, sperm, SLC9C1, NHE10 (sNHE), SLC9C2, NHE11, acrosome, pH regulation

## Abstract

Na^+^/H^+^ exchangers (NHEs) are a family of ion transporters that regulate the pH of various cell compartments across an array of cell types. In eukaryotes, NHEs are encoded by the SLC9 gene family comprising 13 genes. SLC9C2, which encodes the NHE11 protein, is the only one of the SLC9 genes that is essentially uncharacterized. Here, we show that SLC9C2 exhibits testis/sperm-restricted expression in rats and humans, akin to its paralog SLC9C1 (NHE10). Similar to NHE10, NHE11 is predicted to contain an NHE domain, a voltage sensing domain, and finally an intracellular cyclic nucleotide binding domain. An immunofluorescence analysis of testis sections reveals that NHE11 localizes with developing acrosomal granules in spermiogenic cells in both rat and human testes. Most interestingly, NHE11 localizes to the sperm head, likely the plasma membrane overlaying the acrosome, in mature sperm from rats and humans. Therefore, NHE11 is the only known NHE to localize to the acrosomal region of the head in mature sperm cells. The physiological role of NHE11 has yet to be demonstrated but its predicted functional domains and unique localization suggests that it could modulate intracellular pH of the sperm head in response to changes in membrane potential and cyclic nucleotide concentrations that are a result of sperm capacitation events. If NHE11 is shown to be important for male fertility, it will be an attractive target for male contraceptive drugs due to its exclusive testis/sperm-specific expression.

## 1. Introduction

Na^+^/H^+^ exchangers (NHEs) are a branch of the cation/proton antiporter (CPA) superfamily of transporters that are found in the membranes of many cells. Eukaryotic NHEs are evolutionary descendants of prokaryotic Na^+^/H^+^ antiporters (NHAs) [1]. NHEs found in the plasma membrane of higher eukaryotic cells export intracellular protons (H^+^) using the energy stored in the inward directed sodium ion (Na^+^) electrochemical gradient established by the Na,K-ATPase [2]. By exporting protons, NHEs play an important role as regulators of intracellular pH (pH_i_) in many cells [3,4,5,6,7] and thus affect a wide variety of cellular and physiological processes, from brain function [8] to male fertility [9,10,11,12,13,14]. In addition to those found in the plasma membrane, other NHEs display a predominantly intracellular localization and are thought to play roles in regulating pH in intracellular compartments [15].

**Figure 1:**
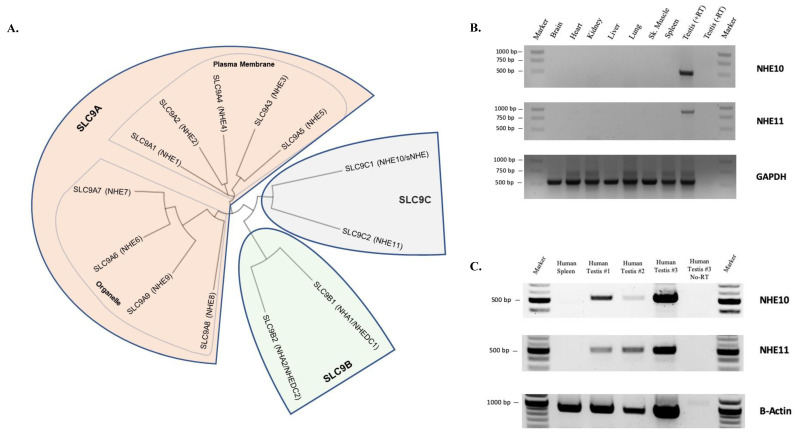
SLC9 gene family and SLC9C2 gene expression. (**A**) Phylogenetic relationships of human SLC9 proteins were calculated using Clustal Omega (https://www.ebi.ac.uk/Tools/msa/clustalo/ (accessed on 7 October 2022), and the tree was generated using Interactive Tree of Life [23]. (**B**) RT-PCR analysis of multiple rat tissues. Both SLC9C1 (NHE10) and SLC9C2 (NHE11) mRNAs are expressed in rat testes but not in any other tissue tested. (**C**) RT-PCR analysis of one human spleen sample and three different human testis samples. NHE10 and NHE11 expression is detected in all three human testis samples but not in the human spleen sample.

NHEs are encoded by the SLC9 family of solute carrier genes. This gene family has been divided into three subfamilies based on sequence homologies: the NHE subfamily (SLC9A1–SLC9A9, which encode NHE1–NHE9), the NHA subfamily (SLC9B1 and SLC9B2, which encode NHA1 and NHA2, also known as NHEDC1 and NHEDC2), and the mammalian sperm-NHE-like subfamily (SLC9C1 and SLC9C2, which encode NHE10, also known as sNHE and the putative NHE11) [6,7] (Figure 1A). Of the 13 SLC9 genes/NHE proteins, 12 have been characterized to some degree, yet nothing has been published about SLC9C2 (NHE11) beyond the existence of the gene in mammalian species. 

There is ample evidence suggesting that NHE activity plays an important role in the regulation of pH_i_ and fertilizing ability in sperm cells. For example, it has been suggested that the alkalization required for the initiation of motility in sea urchin sperm is mediated by NHE activity [16,17,18,19]. It has also been shown that amiloride, an inhibitor of NHE, inhibits rat sperm motility, suggesting the importance of Na^+^ influx and/or H^+^ efflux in mammalian sperm motility [3]. Furthermore, in human sperm, inhibition of NHE activity with a more specific NHE inhibitor, 5-N-ethyl-N-isopropyl amiloride (EIPA), results in the acidification of the cytoplasm in capacitated sperm, suggesting the importance of NHE activity in the regulation of sperm pH_i_ in humans [20]. Finally, inactivating certain NHE genes in mice [9,10,11,12,21] and a mutation in a human NHE gene [14] have been shown to negatively impact male fertility. 

In mature mammalian sperm, there are six NHE protein isoforms known to be expressed: NHE1, NHE5, NHA1, NHA2, and NHE10 [9,11,13,14,22]. According to RNA sequencing evidence, NHE11 (SLC9C2) is also expressed in a testis-restricted manner [7]. Interestingly, in most mammals listed on NCBI’s gene database, SLC9C2 is listed as a protein coding gene. However, in *Mus musculus*, SLC9C2 is instead identified as a pseudogene (designated GM6185). We have thus sought to characterize the expression and localization of the final unstudied SLC9 gene family member, SLC9C2 (NHE11), as a first step in understanding the biological function of this NHE. Our findings suggest that NHE11 could play a role in sperm physiology and male fertility due to its sperm-specific expression. Of potential significance, NHE11 is the only NHE, to our knowledge, to localize to the acrosomal region of the sperm head in mature mammalian sperm.

## 2. Results

We first analyzed SLC9C2 expression in a panel of rat tissues by performing RT-PCR and found that rat SLC9C2 (NHE11) mRNA expression is limited to the testes, just as SLC9C1 (NHE10) expression (Figure 1B). We also performed RT-PCR on RNA from three different human testis samples and found that SLC9C2 mRNA was expressed in each of the testis samples, but not in our negative control sample of a human spleen (Figure 1C). These data are in agreement with the NCBI database (https://www.ncbi.nlm.nih.gov/gene/284525 (accessed on 10 January 2023)) and the Mammalian Reproductive Genetics Database V2 RNA expression database (https://orit.research.bcm.edu/MRGDv2 (accessed on 10 January 2023)), which report testis-limited expression for both rat and human SLC9C2. 

**Figure 2:**
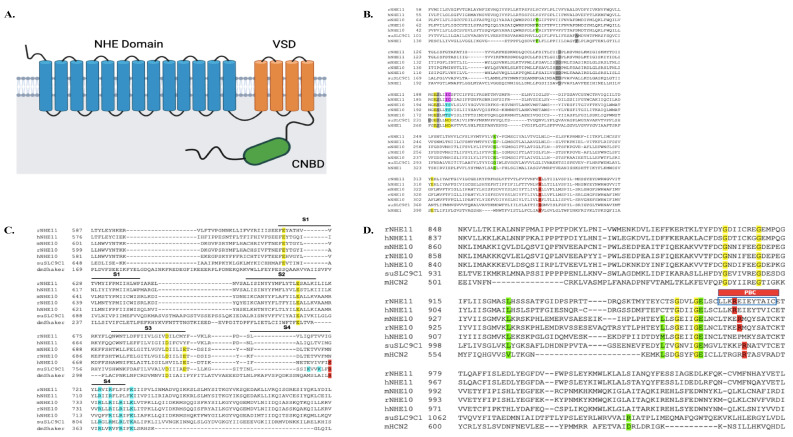
Predicted topology of mammalian NHE11, and sequence alignment comparisons. (**A**) Predicted topology and functional domains of both the rat and human NHE11 protein structure. NHE11 is predicted to be a 17 transmembrane (TM) alpha helical protein with a short extracellular N-terminus and a longer intracellular C-terminus. An NHE domain is predicted within the first thirteen transmembrane domains of NHE11 towards the N-terminus. The VSD is predicted to be contained within the last four transmembrane domains. The long C-terminus of NHE11 is predicted to contain an intracellular CNBD. The protein topology was predicted using the TOPCONS web server [27] and the conserved protein domains were determined with NCBI’s Conserved Domain database [28]. NHE11 topology cartoon created with BioRender.com (accessed on 5 January 2023). (**B**) A portion of the NHE sequence encompassing the predicted transmembrane domains 2–9 of rat and human NHE11 are aligned against mouse, rat, and human NHE10, as well as sea urchin SLC9C1 protein and human NHE1 (hNHE1). Yellow highlighted amino acids are polar amino acids identified as important for NHE exchange in hNHE1 studies [29,30], and are mostly conserved in rat and human NHE11. Note that the ND motif found in hNHE1 and the sea urchin SLC9C1 protein that is conserved amongst electroneutral NHEs [31] is a TS motif in mammalian NHE10 (highlighted in blue) and an IC motif in mammalian NHE11 (highlighted in magenta). Grey highlighted amino acids are residues shown to be important for Na^+^ binding in the sea urchin SLC9C1 protein [19], some of which are conserved in rat and human NHE11. Note that D238 and P239 of hNHE1 are not critical for NHE exchange [30]. Green highlighted amino acids are residues shown to be important for Na^+^ binding to hNHE1 [32,33]; mammalian NHE11 possesses one of the two conserved residues. The red highlighted arginine, when mutated to alanine, abolishes NHE activity of the sea urchin SLC9C1 protein [19] and is conserved in mammalian NHE11s. (**C**) The amino acid sequences predicted to contain the VSD (S1–S4) of rat and human NHE11 are aligned against mouse, rat, and human NHE10, as well as the sea urchin SLC9C1 protein and the drosophila melanogaster Shaker K^+^ channel (dmShaker) VSD. Yellow highlighted amino acids are negatively charged amino acids in S1–S3 and the blue highlighted amino acids are positively charged amino acids in S4, thought to be important for mediating the voltage-gating of the VSD [19]. NHE11 possesses three of the four conserved negatively charged amino acids in the predicted S1–S3 domains of the VSD in the sea urchin SLC9C1 protein. The red highlighted arginine was shown to be important for voltage gating in the sea urchin SLC9C1 protein; when this lysine was mutated to glutamine, the gating currents shifted negatively [19]. Rat and human NHE11 do not contain this specific arginine but do contain three of the seven conserved positively charged residues in the predicted S4 domain of the VSD in the sea urchin SLC9C1 protein. (**D**) The amino acid sequences predicted to contain the CNBD of rat and human NHE11 are aligned against mouse, rat, and human NHE10, as well as the sea urchin SLC9C1 protein and mouse HCN2 (mHCN2) CNBDs. The yellow and green highlighted amino acids are key residues in purine (Val and Leu) and ribofuranose (Gly and Glu) binding [19], most of which are conserved in rat and human NHE11. Notably, the red highlighted arginine is the conserved amino acid in the phosphate binding cassette (PBC) of CNBDs, which rat and human NHE11 also possesses. When this arginine is mutated to glutamine in the sea urchin SLC9C1 protein, the cAMP induced V_1/2_ shift is abolished [19]. The blue box in the PBC identifies the antigenic peptide used to generate our anti-NHE11 antibody (LLKREIEYTAIC).

The full length NHE11 ORF from rat testis cDNA was cloned and sequenced and it is predicted to code for a 1145 amino acid protein. We also sequenced a full-length SLC9C2 clone from a human testis (Transomics Technologies) and found that it contained an ORF predicted to code for a 1124 amino acid protein (Figure S1). Analyses of the potential topology and protein domain conservation of the deduced amino acid sequences of the rat and human NHE11 proteins suggest that the full-length proteins contain three functional domains: an NHE domain within the first 13 transmembrane domains, a voltage sensing domain (VSD) in the last four transmembrane domains, and an intracellular cyclic nucleotide binding domain (CNBD) (Figure 2A). NHE10 (SLC9C1) is also predicted to contain the same three protein domains [9,19]. Many amino acid residues previously suggested to be important for domain function in sea urchin SLC9C1 protein are also conserved in NHE11 (Figure 2B–D) [19]. 

In order to characterize the protein’s transport activity, we attempted to express the full-length rat and human NHE11 proteins in heterologous cell culture systems. However, we were unable to express either NHE11 isoform in cell cultures. To our knowledge, no group has been able to express an unmodified mammalian SLC9C protein in a traditional heterologous cell culture system, despite many previous attempts to express NHE10 [9,19,24,25,26]. The inability to express mammalian NHE10 or NHE11 in cultured cells suggests that these proteins contain amino acid sequences that the transfected cells are unable to process properly, resulting in degradation of the proteins or death of the expressing cells. 

One group has been able to successfully biochemically characterize the sea urchin SLC9C1 protein activity (sea urchins only possess one SLC9C gene) [19]. The sea urchin SLC9C1 protein is a bona fide NHE that exchanges Na^+^ for H^+^ in a 1:1 stoichiometry, and this NHE activity is regulated both by membrane potential (being more active under hyperpolarized conditions) and by cyclic nucleotides (cyclic nucleotide binding enhances NHE activity) [19]. Interestingly, both rat and human NHE11 possess many of the conserved amino acids responsible for NHE activity, voltage sensing, and cyclic nucleotide binding found in the sea urchin SLC9C1 protein (Figure 2B–D). 

**Figure 3:**
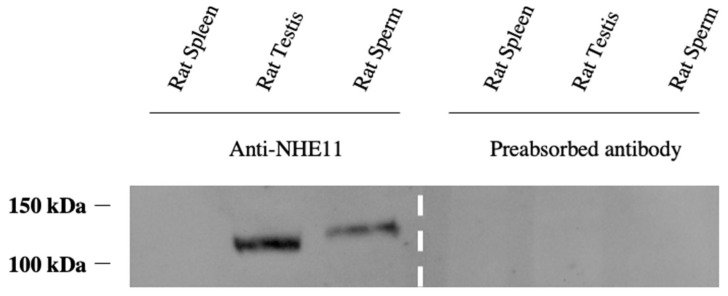
Characterization of the anti-NHE11 polyclonal antibody. Immunoblotting of rat tissue with our affinity-purified anti-NHE11 polyclonal antibody. The blot on the left of the dashed lines shows that this anti-NHE11 antibody recognizes a single band at the predicted size of ~130 kDa in rat testes and sperm, but not in rat spleens. The NHE11 immunoreactive band was not seen if the anti-NHE11 antibody was preabsorbed with the NHE11 antigenic peptide prior to probing the blot, demonstrating the specificity of this antibody. Based on the deduced amino acid sequence, the calculated size for rat NHE11 is 131.5 kDa.

**Figure 4:**
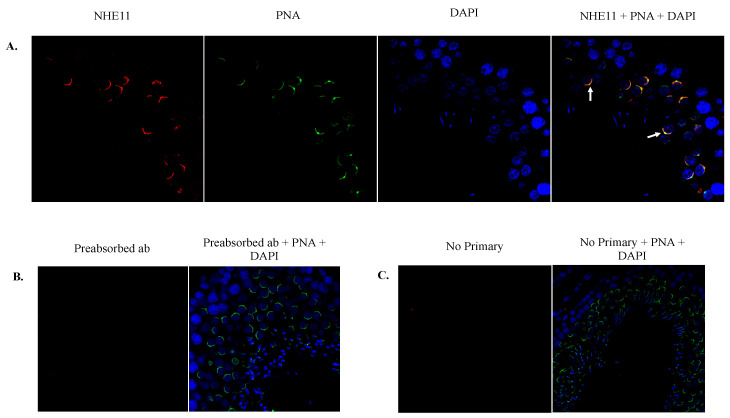
Immunohistochemical localization of NHE11 in rat testis sections. (**A**) Immunolocalization of NHE11 in rat testis sections using our anti-NHE11 antibody and a goat anti-rabbit Alexa 594 secondary antibody (red). PNA-FITC was used to stain the acrosome (green) and DAPI was used to counterstain the nuclei (blue). Note that NHE11 staining of the acrosome is observed in spermiogenic cells. Some spermiogenic cells seem to exhibit NHE11 and acrosomal co-localization (yellow), highlighted by arrows. For controls, (**B**) the anti-NHE11 antibody was preabsorbed with the antigenic NHE11 peptide and subsequently used for immunohistochemistry, or (**C**) the primary antibody was omitted entirely. Images were taken on a Zeiss LSM 710 confocal microscope with a 40× objective.

**Figure 5:**
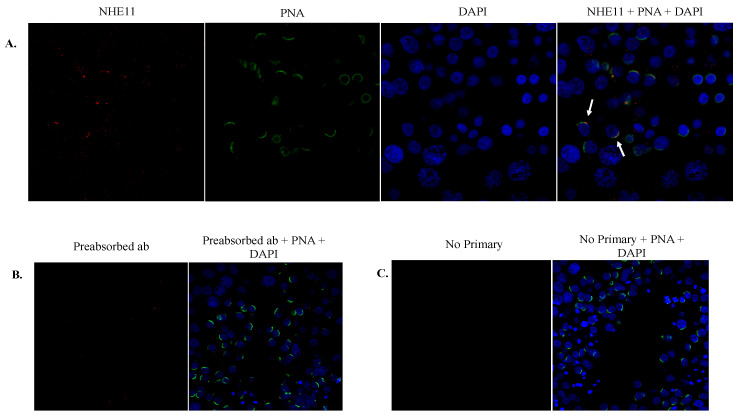
Immunohistochemical localization of NHE11 in human testis sections. (**A**) Immunolocalization of NHE11 in human testis sections using our anti-NHE11 antibody and a goat anti-rabbit Alexa 594 secondary antibody (red). PNA-FITC was used to stain the acrosome (green) and DAPI was used to counterstain the nuclei (blue). Note that NHE11 staining of the acrosome is observed in spermiogenic cells, with a very similar staining pattern to what is seen in the rat testis sections, highlighted by arrows. For controls, (**B**) the anti-NHE11 antibody was preabsorbed with the immunizing NHE11 peptide and subsequently used for immunohistochemistry, or (**C**) the primary antibody was omitted entirely. Images were taken on a Zeiss LSM 710 confocal microscope with a 40× objective.

**Figure 6:**
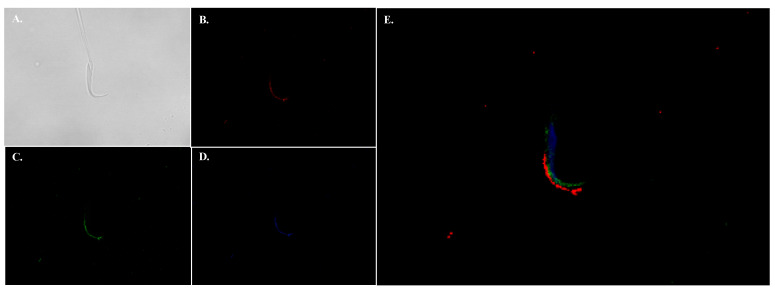
Immunolocalization of NHE11 in mature rat sperm. Immunolocalization of NHE11 in mature rat sperm cell was performed with our anti-NHE11 antibody. (**A**) DIC image of the rat sperm cell. (**B**) Anti-NHE11 immunofluorescence (red). (**C**) PNA-FITC staining of the acrosome (green). (**D**) DAPI staining of cell nucleus (blue). (**E**) Merged image of the three color channels (**B**–**D**). NHE11 appears to localize to the plasma membrane overlaying the acrosomal region in mature rat sperm cells. Images were taken on a Zeiss LSM 710 confocal microscope with a 63× objective.

**Figure 7:**
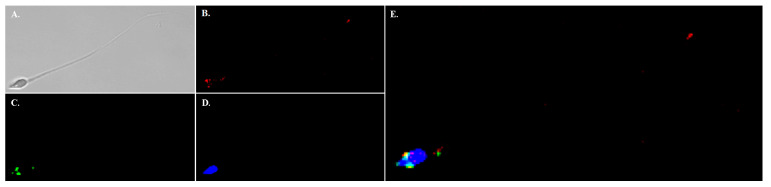
Immunolocalization of NHE11 in mature human sperm. Immunolocalization of NHE11 in mature human sperm was performed with our anti-NHE11 antibody. (**A**) DIC image of the human sperm cell. (**B**) Anti-NHE11 immunofluorescence (red). (**C**) PNA-FITC staining of the acrosome (green). (**D**) DAPI staining of cell nucleus (blue). Note that NHE11 staining is only observed in the sperm head. (**E**) Merged image of the three color channels (**B**–**D**). There may be some colocalization of NHE11 to the acrosomal region (yellow). Note that no NHE11 staining is observed in the sperm flagellum, similar to what is seen in the rat sperm cells. Images were taken on a Zeiss LSM 710 confocal microscope with a 63× objective.

We next generated a polyclonal antibody against an NHE11 peptide (Figure 2D), corresponding to a region of the predicted cyclic nucleotide binding domain (CNBD). This antibody recognizes a single band of ~130 kDa in rat testes and sperm via immunoblotting, corresponding to the 131.5 kDa size of rat NHE11 calculated from the predicted amino acid sequence (Figure 3 and Figure S2). An immunofluorescence analysis of rat and human testis sections reveals NHE11 expression occurs concurrently with the formation of the acrosome in round spermatids and also appears to colocalize with the acrosome throughout spermiogenesis (Figure 4 and Figure 5). Immunofluorescence performed on mature cauda epididymal rat sperm demonstrated NHE11 localization to the plasma membrane of the sperm head, appearing to overlay the acrosome (Figure 6 and Figure S4). Notably, we found no evidence of NHE11 localizing to the sperm flagellum (Figure S3). Human sperm cells exhibited NHE11 localization limited to the sperm head, similar to that seen in rats (Figure 7 and Figure S5). NHE11 protein expression appears to be initiated during spermiogenesis as it is not seen in any earlier developmental stage (or in any non-germ cell type in the testes). Data from the human protein atlas (https://www.proteinatlas.org/ENSG00000162753-SLC9C2 (accessed on 11 January 2023)) support that the NHE11 protein is expressed in spermiogenic cells of the human testes. Additionally, proteomics data from ProteomicsDB (https://www.proteomicsdb.org/protein/64851/expression (accessed on 11 January 2023)) suggest the expression of human NHE11 in only spermatozoon and no other human tissue. Finally, our search of the available mass spectrometry data associated with previous publications revealed evidence of NHE11 protein expression in sperm cells of multiple mammalian species, including donkeys [34], goats [35], boars [36], and humans [37,38]. Taken together, these data suggest that NHE11 is a sperm-specific NHE.

## 3. Discussion

There are five NHEs (NHE1, NHE5, NHA1, NHA2, and NHE10) that have been previously shown to be expressed in mature sperm, and all of them localize primarily to the sperm flagellum. Both NHE1 and NHE5 localize to the sperm midpiece in rats [22]; however, NHE1 has also been shown to localize to both the flagellum and the equatorial region of the sperm head in rams and pigs [39,40]. NHA1 is known to localize to the sperm principal piece in mouse and human sperm and NHA2 is also reported to localize to the principal piece in mice [11]. Finally, NHE10 localizes to the sperm principal piece in mice [9], and has been reported to localize to either the principal piece only [13] or to the entire flagellum [14] in human sperm. 

Unlike what we have shown here for NHE11, there is no definitive evidence that any other NHE localizes to the acrosomal region of the mature mammalian sperm head. There are however two NHEs (NHE3 and NHE8) that have been shown to be critical for acrosomal development. NHE3 (SLC9A3) has been reported to be expressed in mouse testes and to be localized to the acrosome during spermatogenesis through the elongating spermatid stage [21]. NHE3 plays a clear role in acrosome biogenesis because NHE3-deficient mice produce sperm with severely compromised acrosomal structures [21]. Another member of the SLC9A subfamily, NHE8 (SLC9A8), has been reported to localize to mouse germ cells, specifically the forming acrosome [10]. NHE8-deficient male mice are infertile due to severely deformed sperm which exhibit round heads lacking acrosomes and improperly localized mitochondrial sheaths. NHE8 expression was shown to be specifically localized to the acrosome throughout its development during spermatogenesis [10]. Another study reported the expression of NHE8 in Leydig as well as germ cells in mouse and human testis [41]. Importantly, because expression of NHE3 or NHE8 has not been demonstrated in mature sperm in any study [10,21,41], it is unclear whether these NHEs are present in mature sperm or if their expression is only important for acrosome biogenesis and is then lost during sperm maturation. 

Additionally, there are some reports that NHE1 is found in the sperm head as well as the flagellum [39,40]. Strong NHE1 expression was detected in the flagellum with weaker expression in the equatorial region of the sperm head in ram and pig sperm [39,40]. NHE1 had previously been shown to be localized only to the sperm midpiece in rats, not in the sperm head [22]. NHE1 is a ubiquitously expressed NHE that has been suggested to be a “housekeeping” NHE isoform, providing basal pH regulation in all cells [7,42] and its expression in the equatorial region would provide this function to the head of the sperm. Of note, both male and female NHE1 knockout mice are reported to be fertile [8], and our own unpublished data suggest no clear defects in sperm motility in homozygous NHE1 knockout males; therefore, it appears that NHE1 may not be critical for sperm physiology. 

In this study, we have demonstrated that the expression of the SLC9C2 gene is testis-specific and localized the protein product, NHE11, to the acrosomal region of the head in both rat and human sperm. In rat and human spermiogenic cells, NHE11 expression starts concurrently with the formation of the acrosomal granule and there is at least some colocalization between the two (Figure 4 and Figure 5). However, in mature sperm, it appears that NHE11 localizes to the plasma membrane rather than the acrosome (Figure 6 and Figure 7). PNA is known to mainly bind to proteins on the outer acrosomal membrane of mouse, stallion, and human spermatozoa [43,44,45,46] and NHE11 immunostaining is localized adjacent to the PNA-FITC staining, where the plasma membrane would lie (Figure 4). Our results cannot completely rule out that NHE11 may be localized to the outer acrosomal membrane as well as the plasma membrane in mature sperm. It has been shown that the acrosome of human sperm progressively alkalizes during capacitation [47]. Additionally, using calcium channel inhibitors, it was found that human sperm acrosomal alkalization helps trigger Ca^2+^ release from the acrosome and subsequently controls the acrosome reaction [48]. Thus, if some NHE11 does indeed reside in the outer acrosomal membrane, this transporter may have a role in regulating the pH of the acrosomal lumen itself during capacitation.

Even though we found no evidence of NHE11 expression in the rat heart, a very recent study suggests that NHE11 mRNA is expressed in the hearts of both humans and rats [49]. Pérez-Carrillo et al. [49] compared RNA-seq data from healthy human hearts and hearts that had experienced heart failure and found that both NHE1 (SLC9A1) and NHE11 mRNA expression was significantly increased in the heart failure group. ELISAs were used to show that both NHE1 and NHE11 protein expressions were upregulated in the heart failure group (although no validation of either the anti-NHE1 or the anti-NHE11 antibodies used in the ELISA kit was provided). Additionally, rats treated with empagliflozin (a sodium/glucose cotransporter 2 inhibitor used to treat patients with heart failure) showed a decrease in both NHE1 and NHE11 mRNA expression in the heart. Our RT-PCR analysis suggests no expression of NHE11 in rat hearts (Figure 1B) and the available human RNA sequencing data also suggest the same in human hearts (https://www.ncbi.nlm.nih.gov/gene/284525 (accessed on 10 January 2023) and https://orit.research.bcm.edu/MRGDv2 (accessed on 10 January 2023)); therefore, it is unlikely that NHE11 is normally expressed in the heart at a level that is physiologically significant.

There are two members of the SLC9C gene subfamily, SLC9C1 and SLC9C2, both of which share common attributes. First, the SLC9C genes’ expression is restricted to the testes, and both protein products (NHE10 and NHE11) are present predominantly in mature sperm cells. Second, both the NHE10 and NHE11 proteins are predicted to contain an NHE domain within the first 13 transmembrane domains, a voltage sensing domain in the last four transmembrane domains, and an intracellular cyclic nucleotide binding domain at the C-terminus (Figure 2). Although the gene expression patterns and predicted structures of NHE10 and NHE11 are similar, the distinct localizations of the two proteins to different structural compartments of the sperm suggest that these proteins perform individually specialized roles in the context of sperm physiology. NHE10 localizes to the sperm flagellum [13,14], allowing this NHE to facilitate pH regulation in response to various environmental stimuli to influence sperm motility [9,19,24,26]. The exact role that NHE11 plays in sperm is yet to be identified but because of its localization, NHE11 may be regulating pH in the sperm head to facilitate post-capacitation events such as the acrosome reaction or sperm–egg fusion. The generation of an NHE11 knockout model is needed to uncover the specific functional role of NHE11.

Of evolutionary interest, *Strongylocentrotus purpuratus* (sea urchin) only possesses a single SLC9C gene in the genome, which has been designated as SLC9C1. The sea urchin SLC9C1 homolog protein localizes to both the sperm flagellum and the sperm head [19]. Our sequence analysis suggests that the sea urchin SLC9C1 protein is slightly more similar to human NHE11 (24.2% identity and 41.8% similarity) than to human NHE10 (22.2% identity and 40.7% similarity) and therefore it is not possible to unequivocally designate this protein as an NHE10 (SLC9C1) or NHE11 (SLC9C2) homolog (we will refer to the sea urchin isoform as the suSLC9C protein in the rest of this discussion). It is possible that the suSLC9C protein represents a more ancestral form of the protein and that it performs both the functions that NHE10 and NHE11 perform individually in mammalian sperm. If so, this (along with the sequence similarities between NHE10 and NHE11) suggests that the original SLC9C gene duplicated at some point during mammalian evolution and that SLC9C1 and SLC9C2 evolved to produce proteins with separate functions in sperm physiology, potentially dependent, at least in part, on their different localizations in the cell. 

Mammalian sperm undergo a complex physiological maturation process in the female reproductive tract, termed capacitation, that allows the sperm to acquire fertilizing competence. During capacitation, distinct biochemical changes occur in mammalian sperm, which ultimately results in an increased flagellar beating known as hyperactivation, as well as the sperm being able to carry out the acrosome reaction. At the molecular level, capacitated sperm display many important changes, some of which may be directly relevant to the potential function of NHE11. Namely, capacitated sperm experience changes in permeability of various ions, hyperpolarization of the sperm membrane potential, and cAMP-mediated phosphorylation signaling cascades [50]. It has been suggested that the suSLC9C protein functions not in basal pH_i_ regulation, but rather as a signaling molecule that integrates changes in membrane potential and the increase in intracellular cyclic nucleotide concentration associated with capacitation to an increase in intracellular alkalization when the appropriate environmental stimulus is encountered—speract in the case of sea urchin sperm [19]. We suggest that NHE11 may be playing a similar signaling role in mammalian sperm to regulate the pH_i_ of the sperm head in response to hyperpolarization and increased intracellular cAMP concentrations during capacitation.

Characterization of the suSLC9C protein transport activity revealed that it is an electroneutral Na^+^/H^+^ exchanger regulated by membrane voltage and cyclic-nucleotide binding [19]. None of the mammalian NHE10 proteins have yet to be biochemically characterized, likely due to the difficulty of expressing these proteins in heterologous cell culture systems [9,19,24,25,26] and we have encountered similar difficulties in expressing NHE11 in cultured cells. However, some insight into the potential transport function of NHE11 may be gained from the comparison of the amino acid sequences of the mammalian NHE10 and NHE11 proteins to known functional NHEs such as human NHE1 (hNHE1) and the suSLC9C protein. Using the well-characterized hNHE1 protein as a reference for an electroneutral Na^+^/H^+^ exchanger, it is clear that the mammalian NHE11 proteins contain many of the conserved residues important for Na^+^ binding and Na^+^/H^+^ exchange, including the glutamate at E262 and the arginine at R426 in hNHE1 (Figure 2B), suggesting that NHE11 could be a functional NHE. However, the suSLC9C protein and human NHE1 possess an “ND” motif that is conserved in electroneutral Na^+^/H^+^ exchangers, and the aspartate residue in the “ND” motif is highly conserved amongst NHAs and NHEs; the “ND” motif has been shown to be essential for transport activity in multiple NHAs [31]. In contrast, mammalian NHE10 possesses a “TS” motif, while mammalian NHE11 possesses an “IC” motif, suggesting the possibility that these transporters may possess alternative transport activities.

Human sperm have been shown to alkalize both their head and their flagellar principal piece when incubated under capacitating conditions [51]. However, when human sperm are incubated under capacitating conditions in the presence of Cl-GBI, a specific inhibitor of the voltage-gated proton channel Hv1, capacitation-associated alkalization is abolished in the sperm principal piece, but not in the sperm head [51]. These results suggest that since Hv1 localizes to the principal piece in human sperm [52], this proton channel is responsible for the flagellar alkalization during capacitation and leaves open the possibility that NHE11 may have a role in capacitation-associated pH regulation in the human sperm head. 

Evidence for the involvement of an active NHE in the sperm head during capacitation includes that human sperm incubated in Na^+^ deficient media were unable to undergo the progesterone-mediated acrosome reaction [53], and that capacitated human sperm incubated in Na^+^ deficient media or with the NHE-specific inhibitor EIPA displayed a significantly lower pH_i_ [20,53]. These results suggest the possibility of a role for NHE activity in the head of capacitated human sperm involved in regulating the acrosome reaction. NHE11′s localization to the acrosomal region of the sperm head uniquely positions it to mediate these acrosome reaction-dependent pH_i_ changes. 

Similar to NHE10, NHE11 isoforms also possess a putative VSD, comprising four transmembrane domains (S1–S4) that contain multiple charged amino acids thought to be important for modulating ion transport in response to changes in membrane potential. The suSLC9C protein was shown to produce gating currents, suggesting it contains an active VSD. Additionally, the suSLC9C protein mediates voltage-gated Na^+^/H^+^ exchange, being more active under more hyperpolarized conditions. NHE11 possesses three of the four conserved negatively charged amino acids in the predicted S1–S3 domains of the VSD and three of the seven conserved positively charged residues in the predicted S4 domain of the VSD in the suSLC9C protein (Figure 2C) [19], suggesting NHE11 may respond to changes in membrane potential differently to the suSLC9C protein. 

It has previously been shown that human sperm hyperpolarize their membrane potential during capacitation and that this ability of sperm to hyperpolarize under capacitating conditions correlates with successful fertilization [54,55]. The SLO3 K^+^ channel has previously been shown to be essential for mediating the hyperpolarization observed in capacitated mouse sperm [56]. A recent study utilized a novel specific inhibitor of the human SLO3 K^+^ channel, VU0546110, that prevented human sperm from hyperpolarizing and undergoing hyperactivated activity, and prevented the Ca^2+^- or progesterone-induced acrosome reaction [57]. Interestingly, a study using human sperm found no evidence of alkalization of either the sperm head or flagellum in response to valinomycin-induced hyperpolarization [26], suggesting a potential different role of NHE10 and/or NHE11 in human sperm as compared to sea urchin sperm, in which the suSLC9C protein was shown to be responsible for the hyperpolarization-evoked alkalization in response to speract stimulation [19]. However, these human sperm were not incubated under capacitating conditions and the hyperpolarization was induced by the K^+^ ionophore valinomycin [26]; therefore, it is possible that human NHE10 and/or NHE11 are only able to respond to capacitation-induced hyperpolarization. It is also possible that human NHE11’s activity is not regulated by membrane hyperpolarization. 

The sea urchin SLC9C protein possesses an active cyclic nucleotide binding domain which is able to modulate Na^+^/H^+^ exchange in response to an increase in intracellular cAMP concentration similar to that seen in sperm capacitation [19]. Mammalian NHE11 possesses the conserved arginine residue in the predicted phosphate binding cassette of the CNBD, which when mutated in sea urchin SLC9C protein abolished the cAMP-mediated transport activation (Figure 2D). Mammalian NHE11 also possesses many of the conserved residues likely important for the purine and ribofuranose of the cyclic nucleotide binding to the CNBD (Figure 2D) [19,58]. Whether the mammalian NHE11 CNBD can actually bind to cyclic nucleotides such as cAMP and fine-tune NHE activity has yet to be determined. 

As a secondary messenger molecule, cAMP plays an important role in sperm capacitation (and ultimately fertilization), and an increase in intracellular cAMP is one of the first events of capacitation [59,60,61]. The enzyme that produces the majority of cAMP in sperm is the soluble adenylyl cyclase (sAC, encoded by the ADCY10 gene), a protein which has been shown to be essential for sperm motility and fertility [62,63]. Interestingly, in mouse sperm, the NHE10 and sAC proteins associate with one another and their co-expression appears to be essential for sperm fertility [24]. NHE10-deficient mouse sperm lack expression of the full-length sAC protein isoform, which in turn results in severely diminished bicarbonate stimulated cAMP synthesis, resulting in the infertility phenotype. Coimmunoprecipitation experiments revealed that NHE10 and suSLC9C proteins both physically interact with sAC in mouse [24] and sea urchin sperm [64]. sAC has been shown to localize to the midpiece in mouse sperm [63] and to both the head and flagellum in sea urchin sperm [65]. Where sAC localizes in rat and human sperm has not yet been identified, but it will be interesting to see if NHE11 also physically interacts with sAC in mammals to form a protein complex either in mature sperm or during spermiogenesis. The proximity of sAC to NHE10 (and potentially NHE11) would allow the cAMP synthesized by sAC to rapidly mediate NHE10 and/or NHE11 exchange activity in response to capacitation events. 

In addition to sAC, several transmembrane adenylyl cyclase (tmAC) isoforms have been shown to localize to the head of mouse sperm [66,67], along with stimulatory Gα_s_ protein subunits [68,69]. Additionally, there is evidence of compartmentalized tmAC adenylyl cyclase activity after capacitation in the mouse sperm head that was shown to be able to induce the acrosome reaction independent of sAC activity in the sperm flagellum [68,70]. Inactivation of the ADCY3 gene in mice results in subfertile male mice due to defects in sperm motility and the acrosome reaction [67,71]. There is also evidence of Gα_s_ protein subunit [69] and tmAC isoform 3 (ADCY3) expression in the head of human sperm [72]. The expression of these proteins could provide a highly localized source for cAMP production in the sperm head to modulate human NHE11 activity. 

The exact function of NHE11 remains yet to be determined, but its unique localization to the acrosomal region of the sperm head suggests it may be playing an unrecognized role in sperm physiology. Due to its similarity to NHE10 and to its localization, NHE11 could be involved in regulating the pH of the acrosomal lumen and/or the sperm head to facilitate post-capacitation events (e.g., the acrosome reaction or sperm-egg fusion) in response to hyperpolarization or intracellular cAMP levels. More work is needed to identify its exact function and also determine if NHE11 is indeed a bona fide sodium hydrogen exchanger that is regulated by membrane potential and cyclic nucleotides such as the sea urchin SLC9C protein. If NHE11 is proven to be critical for male fertility, its exclusive testis/sperm-specific expression makes it a logical target against which male-specific contraceptive drugs could be developed.

## 4. Materials and Methods

### 4.1. Tissue Collection

Tissues were collected from six sexually mature male Long–Evans rats (3–6 months in age) euthanized by CO_2_ asphyxiation followed by cervical dislocation. The tissues collected were brain, heart, kidney, liver, lung, skeletal muscle (gastrocnemius), spleen, testis, and cauda epididymal sperm cells. Frozen and formalin-fixed paraffin-embedded human tissues were acquired from the Cooperative Human Tissue Network and consisted of human testis samples from three different adult men and a human spleen sample from one adult male. 

### 4.2. RT-PCR

Frozen rat and human tissues were homogenized by sonication in TRI Reagent (Zymo Research, Irvine, CA, USA) and RNA was isolated using the Direct-zol RNA MiniPrep Plus kit (Zymo Research). Five hundred nanograms of RNA was converted to cDNA with M-MuLV Reverse Transcriptase (New England BioLabs, Ipswich, MA, USA) according to the manufacturer’s recommendations using an oligo-dT primer in order to preferentially convert poly-adenylated RNA transcripts. The diluted cDNA (1:10) was used as a template for PCR reactions with the primers detailed in Table 1. Q5 High Fidelity DNA polymerase (New England BioLabs) was used for amplification of the rat NHE11 ORF. All other PCR reactions were performed with DreamTaq DNA polymerase (ThermoFisher, Waltham, MA, USA). The PCR product of the full-length ORF of rat NHE11 was gel purified using a gel extraction kit (IBI Scientific, Dubuque, IA, USA) and then cloned into the pMiniT 2.0 vector using the NEB PCR Cloning Kit (New England BioLabs). The full-length human NHE11 cDNA clone was purchased from Transomic Technologies (Clone ID: BC042592-seq). The cDNAs were Sanger sequenced (EuroFins Genomics, Louisville, KY, USA). 

### 4.3. Generation of Anti-NHE11 Specific Polyclonal Antibody

Polyclonal antiserum against NHE11 was generated in rabbits using a synthetic 12 amino acid peptide corresponding to amino acids 965–976 of the deduced rat NHE11 amino acid sequence. Briefly, in order to produce the NHE11 polyclonal antibody, the unique peptide sequence (NH_2_-LLKREIEYTAIC-COOH) was synthesized by the Antibody Research Corporation (Saint Peters, MO, USA), where it was conjugated to keyhole limpet hemocyanin (KLH) and used to immunize rabbits. Our NHE11 peptide (above) was conjugated to a column containing a 6% crosslinked agarose support using a SulfoLink Immobilization Kit for Peptides (ThermoFisher). Sera from the injected rabbits were then affinity-purified using this column. The concentration of purified NHE11 antibody was quantified spectrophotometrically at 280 nm.

### 4.4. Immunoblotting

Snap frozen rat tissues resuspended in Transmembrane Protein Extraction Reagent (FIVEphoton Biochemicals, San Diego, CA, USA) containing 1× Halt Protease Inhibitor Cocktail (ThermoFisher) and 1 mM EDTA (ThermoFisher) were homogenized with a Dounce homogenizer on ice. Rat sperm pellets were homogenized in the same lysis buffer as above by sonication. The rat tissues and sperm lysates were cleared by centrifugation and then the supernatants were stored at −80 °C until used for immunoblotting. Protein concentrations of the tissue lysates were estimated using the Pierce BCA Protein Assay Kit (ThermoFisher). Tissue and sperm lysates (30 µg) were mixed into 1× Laemmli buffer (Bio-Rad, Hercules, CA, USA) containing BME (Bio-Rad) according to the manufacturer’s instructions and then incubated at 37 °C for 30 min. The denatured and reduced samples were resolved on a 10% Mini-Protean TGX Stain-Free Gel (Bio-Rad) and then transferred to an Immobilon-P PVDF membrane (Millipore Corporation, Burlington, MA, USA) using a Trans-Blot Turbo Transfer System (Bio-Rad). 

For the NHE11 immunoblotting, a SuperSignal Western Blot Enhancer (ThermoFisher) was used to amplify the NHE11 signal. The manufacturer recommended protocol was used, which was in brief: The blot was washed with ddH_2_O following transfer and then incubated in the Antigen Pretreatment Solution. The blot was then washed with ddH_2_O and then blocked in EveryBlot Blocking Buffer (Bio-Rad). The blot was then washed with wash buffer (0.1% TBS-Tween 20) prior to probing with our anti-NHE11 antibody at a concentration of 0.5 µg/mL diluted in the supplied Primary Antibody Diluent overnight at 4 °C. The following day, the blot was washed with wash buffer and then probed with goat anti-rabbit horseradish peroxidase conjugated secondary antibody (Jackson ImmunoResearch, West Grove, PA, USA) at a 1:10,000 concentration in 5% non-fat dry milk in wash buffer for 30 min at room temperature. Then, the blot was washed with wash buffer and finally, SuperSignal West Pico PLUS Chemiluminescent Substrate (ThermoFisher) was added to the blot. The chemiluminescent signal was analyzed using a ChemiDoc MP imaging system (Bio-Rad). For blocking peptide experiments, our anti-NHE11 antibody was preabsorbed with five times excess (by weight) of the NHE11 peptide used for antibody generation by rocking at room temperature for 1 h in the Primary Antibody Diluent before probing. 

### 4.5. Preparation of Testes Sections for Immunofluorescence Analysis

Dissected rat testes were fixed in 10% formalin overnight at room temperature with rocking. Then, the fixed rat testes were serially dehydrated, cleared in xylene, and embedded in paraffin. Paraffin-embedded human testis blocks were acquired from the Cooperative Human Tissue Network. Both rat and human testis blocks were cut into 5 µm sections and attached to glass slides. These sections were deparaffinized and rehydrated and then heat-induced antigen retrieval was performed in 1× Citrate Buffer (pH 6.0) (Invitrogen, Waltham, MA, USA). 

### 4.6. Preparation of Sperm for Immunofluorescence Analysis

Rat cauda epididymal sperm and human sperm were pelleted and washed in PBS, and then the sperm suspensions were spread on glass coverslips placed on a slide warmer set at 37 °C to airdry. Once dry, the sperm cells were fixed in 10% formalin for 10 min at room temperature and then were washed with PBS. 

### 4.7. Immunofluorescence Analysis

Testis sections were permeabilized in 0.3% Triton X-100 in PBS and then blocked in 10% normal goat serum (NGS) in 0.3% Triton X-100 in PBS. The sections were then incubated overnight at 4 °C in the above blocking solution containing 20 µg/mL anti-NHE11 antibody. The next day, the sections were washed in 0.1% PBS-Tween 20 solution (PBST) and then incubated in the above blocking solution containing IgG (H + L) Cross-Adsorbed Goat anti-Rabbit Alexa 594 (Invitrogen) (1:100), Lectin from *Arachis hypogaea* FITC-conjugated (PNA-FITC) (Sigma-Aldrich, St. Louis, MO, USA) (50 µg/mL), 4′,6-Diamidino-2-Phenylindole, and Dilactate (DAPI) (Invitrogen) (1:1000) for 2 h at room temperature in the dark. Then, the sections were washed in PBST, and then the slides were mounted in VECTASHIELD Antifade Mounting Medium (Vector Laboratories, Newark, CA, USA) and the coverslips were sealed. 

Fixed sperm were permeabilized in 0.2% Triton X-100 at room temperature for 10 min. Then, the sperm were washed in PBST and then the coverslips were blocked in Antigen Signal Enhancer (ASE) blocking solution (50 mM glycine, 0.05% Tween 20, 0.1% Triton X-100, and 10% NGS in PBS) [73] for 30 min at room temperature. The coverslips containing the fixed sperm were incubated overnight at 4 °C in ASE blocking solution containing 20 µg/mL of our anti-NHE11 antibody. The next day, the coverslips were washed with PBST and then incubated in ASE blocking solution containing IgG (H + L) Cross-Adsorbed Goat anti-Rabbit Alexa 594 (Invitrogen) (1:100), PNA-FITC (50 µg/mL), and DAPI (1:1000) for 2 h at room temperature in the dark. The coverslips were washed in PBST, and the slides were mounted in VECTASHIELD Antifade Mounting Medium and then the coverslips were sealed to glass slides.

For blocking peptide experiments, our anti-NHE11 antibody was preabsorbed with NHE11 peptide used for antibody generation before probing the samples. For the no primary antibody experiments, the samples were incubated in blocking solution without the primary antibody overnight at 4 °C before incubation with the secondary antibody. All testis and sperm samples were imaged on an LSM 710 Confocal Microscope (Zeiss, Jena, Germany).

## Figures and Tables

**Table 1:** Primers used in this study for RT-PCR analysis. (S) is a sense primer and (AS) is an antisense primer.

Species	Gene Targeted	Primer Sequence	Amplicon Size (bp)
Rat	GAPDH	5′-TGGTGAAGGTCGGTGTGAACG-3′ (S)	533
		5′-GGCATGGACTGTGGTCATGAG-3′ (AS)	
Rat	NHE10	5′-CAGAATATCTGCTCAGCGGG-3′ (S)	508
		5′-GGTGGGTTGTACTTTTTTACATTCC-3′ (AS)	
Rat	NHE11	5′-GACGATCCGCAACGTGATTG-3′ (S)	914
		5′-TTCGAAGACCAGTGCTGACC-3′ (AS)	
Rat	NHE11 FL ORF	5′-ATGTCGAACAACAGTTTTTC-3′ (S)	3435
		5′-ATATGTCTTTCTAAAGAGGT-3′ (AS)	
Human	ACTB	5′-CACTGGCATCGTGATGGACT-3′ (S)	952
		5′-CGCATCTCATATTTGGAATGACTAT-3′ (AS)	
Human	NHE10	5′-TCTGCCACCTGCAAAACTGT-3′ (S)	541
		5′-CACTGTGTGCTCCATCCTCC-3′ (AS)	
Human	NHE11	5′-CCAATGTCATGGCCTCAGTCA-3′ (S)	530
		5′-CCAAACTGGGAAGGGGAGAAT-3′ (AS)	

## Data Availability

The data supporting the findings of this study are available within this article and its Supplementary Materials.

## Supplementary Materials

The supporting information can be downloaded at: https://www.mdpi.com/article/10.3390/ijms24065329/s1.
