# Supplementary material for: The SLC9C2 Gene Product (Na+/H+ Exchanger Isoform 11; NHE11) Is a Testis-Specific Protein Localized to the Head of Mature Mammalian Sperm
Source: Int J Mol Sci. 2023 Mar 10;24(6):5329. doi: 10.3390/ijms24065329 (PMC10049371; doi:10.3390/ijms24065329)
Supplement: Supplementary file 1 [file ijms-24-05329-s001.zip › ijms-2239717-supplementary.pdf]

The SLC9C2 gene product (Na^+/H^+ exchanger isoform 11; NHE11) is a testis-specific protein localized to the head of mature mammalian sperm

Supplemental Material

```
rNHE11    1  MSNNSFSFWVARNDNKPDLLCGRPADYFVVQKHFTILICATIVLGGFSRMVLKNSEVFVMIILSVSGFV
hNHE11    1  MSS---YFWAQNESNRPDLLCGQPADYLVEEKHFTTLVCFIVVLGGLLKMCLKNCEVIVLTILSLSGFV

rNHE11   70  TGRLAYNFIEVHQIVYPLLKTPSFSLYCYFLPLIVFVAALDVDFYIVKNVFWQVLLTGLISFGTAFATI
hNHE11   67  IGHMAYNSVEVHQIVYPLLRTSSFSLYSYFSPLIIFMVALDVEFYTLKKMFWQVLLTGLISFSTASIII

rNHE11  139  SYVVLKFHEDKWDLQCCLLFSITLCIIDPLHSVNSLKMIGISKMYTDIIRGESLIICSFTCIFFGIFRG
hNHE11  136  GYVVIKFNKDSWDLQSCLLFSITLGIIDPLRSVNSLKTIGISKIYIDLIRGESLIICSIASIFFGNFRG

rNHE11  208  HTTNVSMFRELHVIIGLSFDIFGSAVCGYWCTRVIQIILTDLFSNTLTNVVLCFSLVYMTFYLVEYFGM
hNHE11  205  NRIHFSIFRDLHVGIELSYDILGSIIFGYWCAKIIQCILADVFSNMLTNIILCFSMVYMTFYIVEFLGM

rNHE11  277  SGIVALVTVGLNLDSLSFKPRMEFIITKFLIMCSSVYEHLIYAFFGIVIGCGEIKYFRFHSLVFTVTLF
hNHE11  274  SGTLALAAVGLNLDSLTFKPKIELVITKFLRIFSSVYEHLIYAFFGIVIGCGELSHYEFHTIPFIFILF

rNHE11  346  VTVNFVRLLTIILVSPILMHSSYEYDWRWGVVITWSGIRGVFSLLLAPDIYNFSEYKLAAPHMCIFYIQ
hNHE11  343  TTVNLVRLLTILLVSPILMHSNYEYNWRWGVVITWSGIKGVFNLLWAPDVYNLAERKVEVPQMFILYVQ

rNHE11  415  ALSILTTGINSHMMLRSAKTLGLCALSLPRQMAVRNATEHIQEIIRNTITLFKTEKILTNVNWNVVEEK
hNHE11  412  VISLLTMGINSYVMTQSARKLDLCVLSLPRQMILQNATQHIQEIVQNTITLFKTEKILTNVNWTLVEDK

rNHE11  484  AKIEYNPILKQARTVPLAHISHGP REESPTDEVLIEEARLHVAIIQMSSFEKQCNDGILGVEAARILI
hNHE11  481  TRIEY---------IPFSHVSHNDMKTESTTDEALMEEARLHVAAIQMSSFEKQRNNGILEIEAARILI

rNHE11  552  GATKSYCPIHGKFMSISDVSTYVRARSWLIKFKNMLTYLEYHKERVLFTVPGNNKLLIFVYRIIISEEF
hNHE11  541  GAAKCYYSIQGKFMSIYDVSTYMRTRSWLIKFKNVLTFLEYCIEKIHFIPPESNTFLTFIFHIVFSEEF

rNHE11  621  EYATHVVTVMYIFPMIIHLWPIARELNVSALISINYYYMFLYTLESALKILILKRKYFLQNWNTLDFFI
hNHE11  610  EYTGQIINLIYIYPMIIHLWPMARGLNVSALISINYYFMFLYVLESTLKIIILKRKYFQQCWNTLEFFI

rNHE11  690  IVLGIVDILCMYFVKLRPDSLVLIQFTVVIGYLRVIRFLPIFKIIIPVLINMADVQIKKSLSLMYSITK
hNHE11  679  LVIGIIDIFCVYFVKLRPDNLALIQLTVIMGYLRIIRFLPLFKIIVPILIRIADVQIKKRLSLMYSITK

rNHE11  759  GYVKSQEDAKLLVRQISGRESIYQKLYDILERNKREAIRELGLIEHEGRDVVIALKTKQTIRNVIAKAL
hNHE11  748  GYIKSQEDAKLLIKQIAVCESIYQKLCEILETNKQDAVKELVLMEHEGRDVVIALKTKQAIRNVIAKAL

rNHE11  828  KNLTFLWSRGIIDKHEGIEMNKVLLTKIKALNNFPMAIPPPTPDKYLPNIVWMENKDVLIEFFKERTKL
hNHE11  817  KNLTFLCSRGIIDKHEVIEINKVLLKKLKALNNFPKAIPPPTPDIYLHNIIWLEGKDVLIDFFKERAKL

rNHE11  897  TYFDYGDIICREGEMPQGIFLIISGMASLHSSSATFGIDPSPRTTDRQSKTMYTEYCTSGDVLGELSCL
hNHE11  886  ACFDSGDTICKGGEMPQGIYLIISGMAILHSLSPTFGIESNQR CDRGSSDMFTEFCTTGDIIGELSCL

rNHE11  966  LKREIEYTAICETTLQAFFISLEDLYEGFDVFWPSLEYKMWLKLALSIANQYFESSIAGEDLKFQKCVM
hNHE11  954  LKREIEYTVICETSLQACFISLEDLYEGFDAFWPSLEYKIWLKLALSTAYQYFESSLIDEDLRFQNCVM

rNHE11 1035  FNHAYVETLSSYNEMTLNHVNMKLIILVYGNVINSKTEETHVAPCIIPKSCEQIQGISDVSKLLVIPTS
hNHE11 1023  FNQAYVETLSSYSDMIIDNMTMKFVIIVYGSVIDTKTEEPYFAPCIIPTTCEQVQGTSDLSKLLIIQAS

rNHE11 1104  EPVKKDATSHDV--VSTGLRTTKKDYSWKRKDEFEGGHLFRKTY
hNHE11 1092  ELTQRNSNTNVMASVNTVFEQPGKNINGRQKMS-----------
```

Supplementary Figure S1. Rat and human NHE11 full-length amino acid sequence alignment.

The deduced rat and human NHE11 amino acid sequences from the cloned cDNAs were aligned. Rat and human NHE11 exhibit a 67.10% identity, 78.62% similarity to each other. Black font indicates identical amino acids.

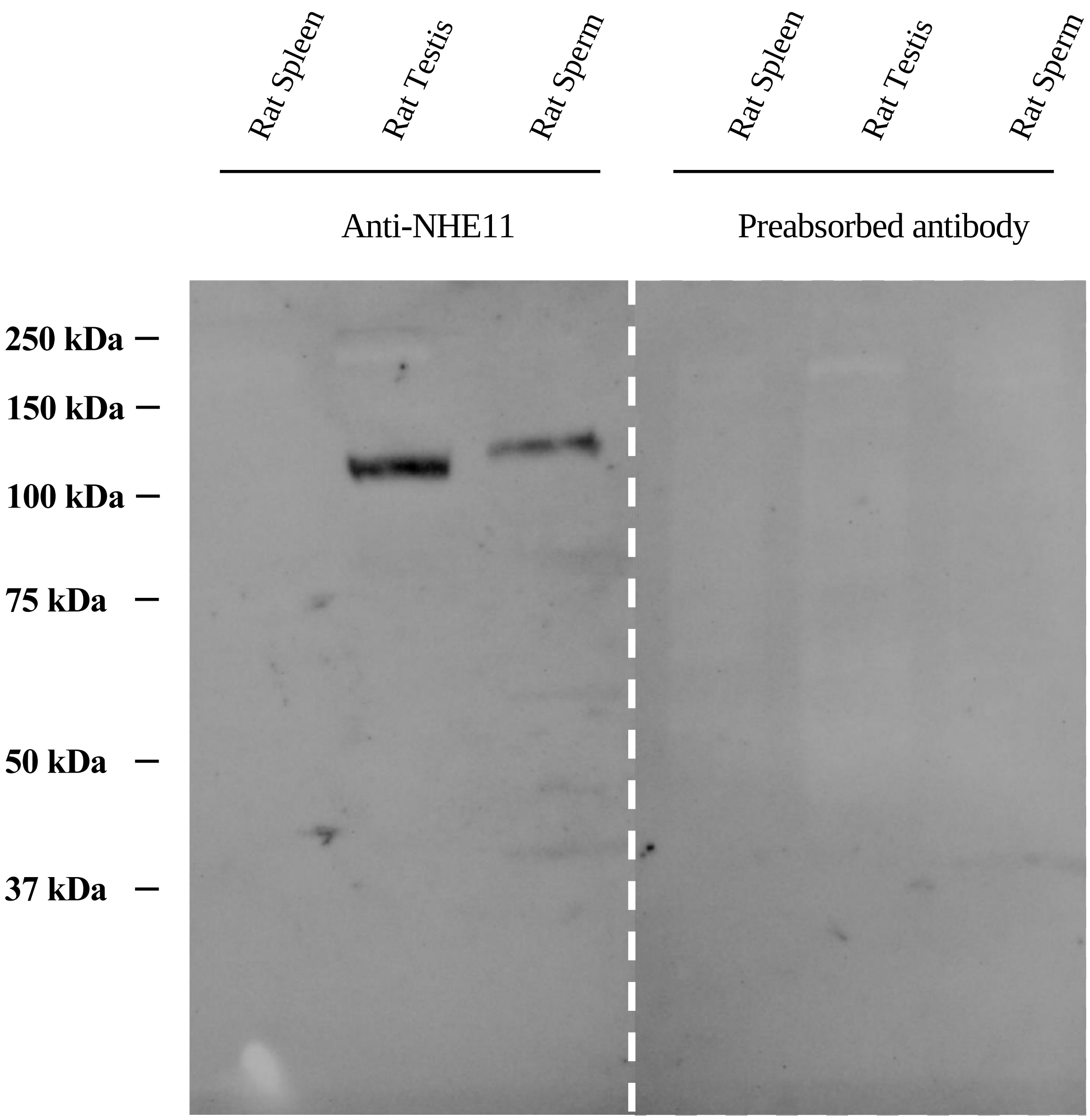

Supplementary Figure S2. Anti-NHE11 antibody specificity.
The full immunoblot from Figure 3 is shown here uncropped. A single band in the rat testis and sperm lanes are shown corresponding to the predicted size of NHE11. The NHE11 immunoreactive band was not seen if the anti-NHE11 antibody was preabsorbed with the NHE11 antigenic peptide prior to probing the blot, demonstrating the specificity of this anti-NHE11 antibody and that the immunoreactive band is not a result of non-specific secondary antibody interactions.

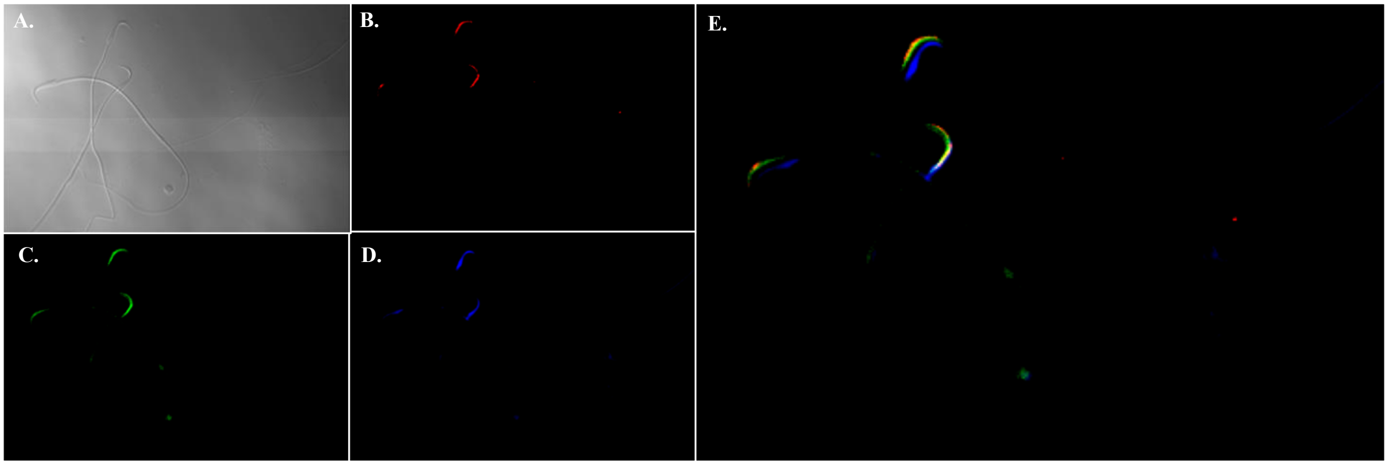

Supplementary Figure S3. Anti-NHE11 immunocytochemistry on rat sperm cells demonstrates that NHE11 does not localize to the sperm flagellum.
Immunolocalization of NHE11 in mature rat sperm cells was performed with our anti-NHE11 antibody. A) DIC image of rat sperm cells. B) Anti-NHE11 immunofluorescence (red). C) PNA-FITC staining of the acrosome (green). D) DAPI staining of cell nucleus (blue). E) Merged image of the three color channels (B, C, and D). NHE11 appears to localize to the plasma membrane overlaying the acrosomal region in mature rat sperm cells. There may be some colocalization of NHE11 to the acrosomal region (yellow). Note that there is no observed NHE11 staining of the rat sperm flagellum.

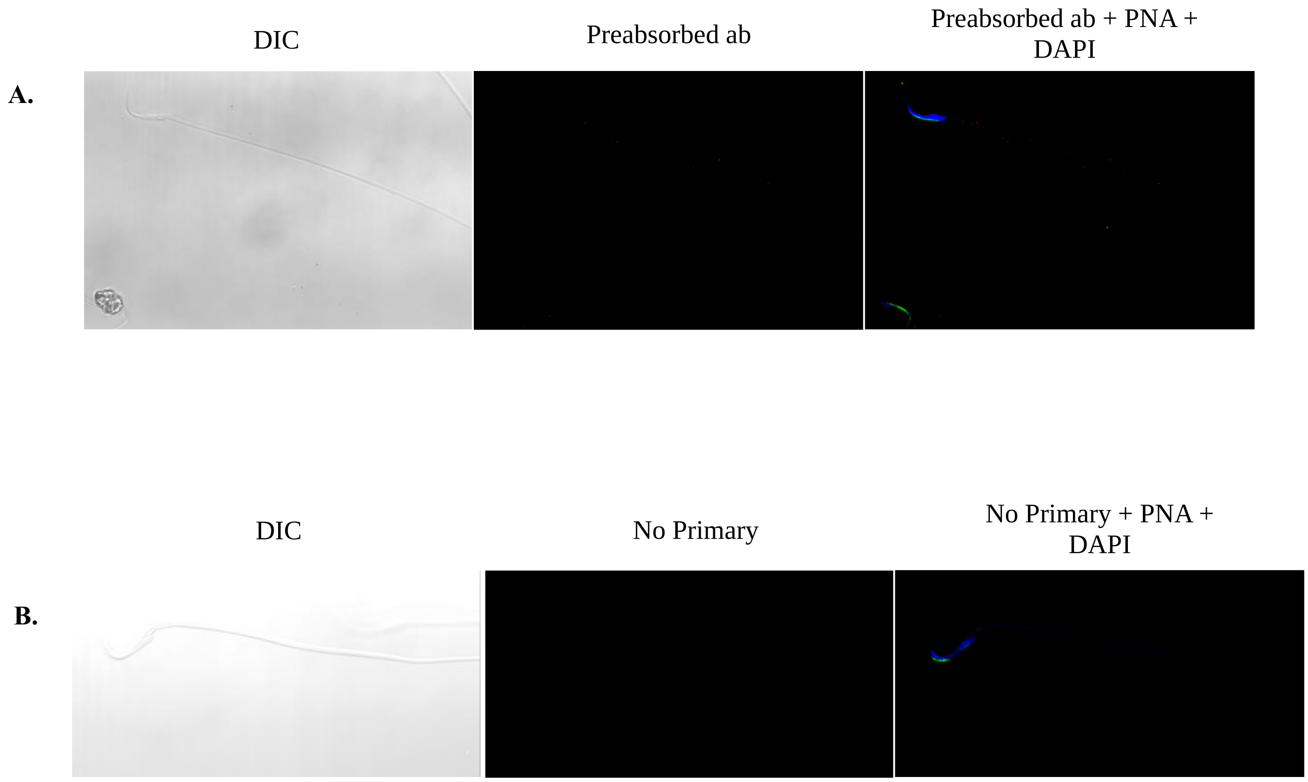

Supplementary Figure S4. Negative controls for rat sperm immunocytochemistry experiments.

For negative controls the rat sperm acrosomes and nuclei were stained with PNA-FITC and DAPI respectively, A) the anti-NHE11 antibody was preabsorbed with the antigenic NHE11 peptide and subsequently used for immunocytochemistry or B) the primary antibody was omitted entirely.

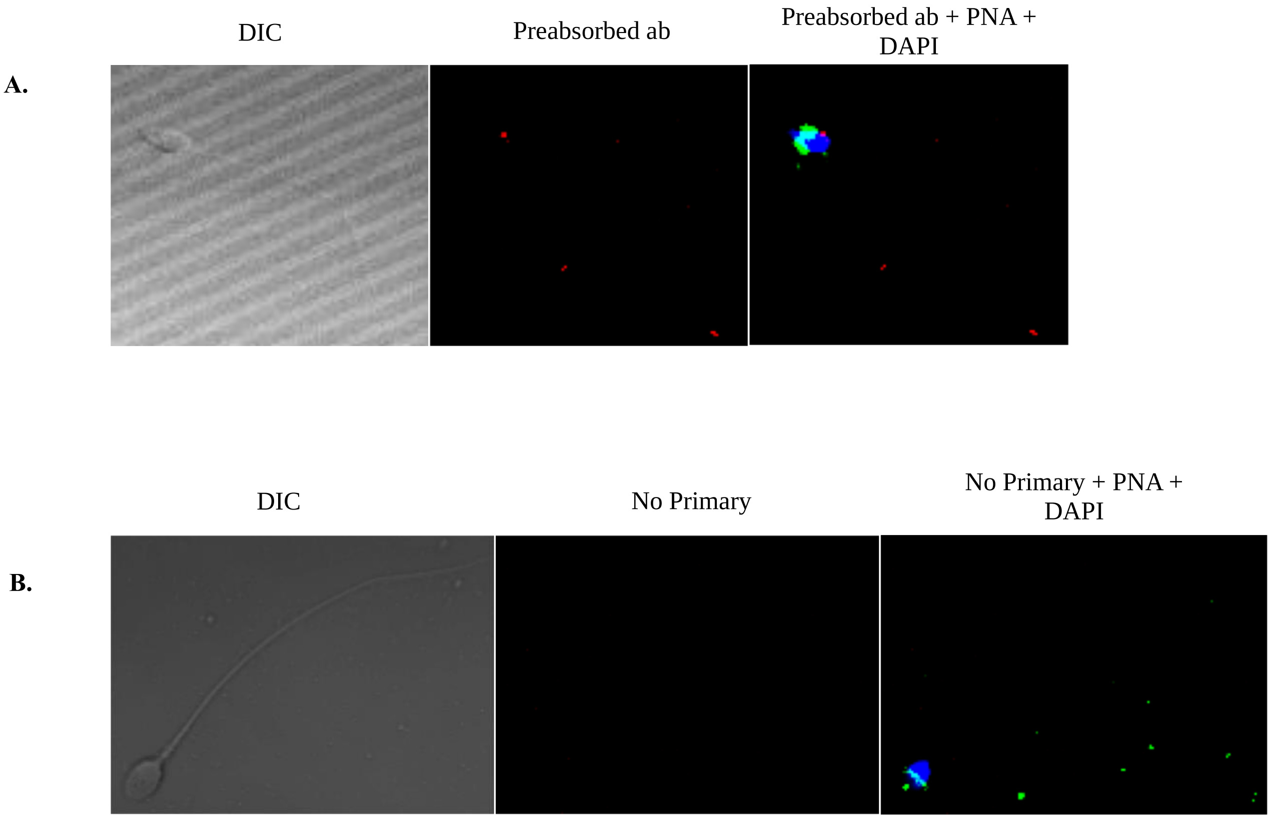

Supplementary Figure S5. Negative controls for human sperm immunocytochemistry experiments.

For negative controls the human sperm acrosomes and nuclei were stained with PNA-FITC and DAPI respectively, A) the anti-NHE11 antibody was preabsorbed with the immunizing NHE11 peptide and subsequently used for immunocytochemistry or B) the primary antibody was omitted entirely.
